# The mechanisms underlying the influence of perceived pressure on doctoral students’ learning engagement: a serial mediation of social support and academic self-efficacy

**DOI:** 10.3389/fpsyg.2026.1747603

**Published:** 2026-01-27

**Authors:** Jindan Zhang, Peibo Wu, Wenxin Chen, Fang Fang

**Affiliations:** 1 Office of Academic Affairs, Ningbo University of Technology, Ningbo, China; 2 Mental Health Education Center, Henan University of Engineering, Zhengzhou, China; 3 Institute of Education, Xiamen University, Xiamen, China

**Keywords:** academic self-efficacy, doctoral students, learning engagement, perceived pressure, social support

## Abstract

**Purpose:**

This study aims to examine how perceived pressure influences doctoral students’ learning engagement, specifically analyzing the serial mediating roles of social support and academic self-efficacy.

**Methods:**

A cross-sectional questionnaire survey was conducted with a sample of 432 Chinese doctoral students, utilizing the Perceived Pressure Scale, the Social Support Scale, the General Academic Self-Efficacy Scale, and the Classroom Engagement Scale.

**Results:**

Perceived pressure was positively associated with doctoral students’ learning engagement via social support (*β* = 0.065, 95% CI [0.036, 0.100]), and also through academic self-efficacy (*β* = 0.075, 95% CI [0.068, 0.173]). Additionally, a significant serial mediation pathway was identified—perceived pressure → social support → academic self-efficacy → learning engagement (*β* = 0.015, 95% CI [0.010, 0.041]). Social support and academic self-efficacy served as serial mediators in the relationship between perceived pressure and learning engagement.

**Discussion:**

Perceived pressure among doctoral students shows a positive direct association and multiple indirect associations with learning engagement. From a Job Demands–Resources (JD-R) perspective, these findings suggest that perceived pressure may function as a challenge demand when adequate resources are available, and that social support and academic self-efficacy jointly facilitate engagement through a sequential resource pathway. While focusing on doctoral students’ learning engagement, higher education institutions should emphasize the development of robust social support systems and the enhancement of academic self-efficacy to assist doctoral students in transforming stress into a sustained driving force for academic development.

## 1 Introduction

Doctoral students generate new knowledge through their training and thereby advance disciplinary development (Casey et al., 2023). They are the main force of national and social progress, and their learning engagement constitutes a positive, fulfilling, work-related psychological state. This engagement strengthens doctoral students’ psychological resilience (McCray and Joseph-Richard, 2020) and reduces anxiety and burnout. Deep academic investment fosters critical thinking, problem-solving, communication, and collaboration skills (McAlpine and Amundsen, 2011), enables mastery of advanced research methods, and ultimately yields high-quality scientific outputs (Yang and Yuan, 2020; Desie and Tefera, 2017).

Doctoral students often confront challenges such as mentorship contradictions (Mavrogalou-Foti et al., 2024), academic–life imbalance (Girard et al., 2024), and bearing certain perceived pressure (Casey et al., 2023; Mahsood et al., 2025). These issues are especially acute in China, which hosts the world’s largest doctoral education system but where doctoral students report greater stress than their international peers (Woolston and O’Meara, 2019). First, many Chinese doctoral students face strict graduation requirements that mandate publishing a specified number of articles in high-impact journals. This “publish-or-perish” culture fosters persistent anxiety. Second, doctoral supervisors in China often hold authority over academic progression and stipend allocation, creating power imbalances. Finally, relatively low financial stipends received by many Chinese doctoral students exacerbate their work-life conflict (Woolston and O’Meara, 2019).

Perceived pressure is a widely used concept in psychology and medicine (Goodnite, 2013), denoting an individual’s feelings or thoughts about the pressure experienced at a particular moment or over a specific period (Weerasinghe, 2024). The mental health of doctoral students directly affects the quality of talent supply and the overall academic productivity of the research sector (Podsakoff et al., 2007). Prior research shows that when pressure remains within an individual’s coping capacity, it is appraised as a challenge and can stimulate intrinsic motivation; when it exceeds that capacity, it undermines physical and mental health and development (Horan et al., 2020). As Podsakoff et al. (2007) noted, doctoral students who judge academic pressure to be acceptable may experience increased learning motivation and improved research efficiency. However, prolonged or chronic high perceived pressure leads students to reduce investment in tasks requiring heavy cognitive load and deep thinking as a defensive response to internal or external psychological threats (Byrom et al., 2020).

This study adopts the Job Demands-Resources (JD-R) model (Demerouti et al., 2001) as its theoretical framework. According to this model, the environment consists of ‘job demands’ (e.g., perceived pressure) and ‘job resources’ (e.g., social support, academic self-efficacy), which jointly determine the level of doctoral students’ learning engagement. However, existing literature has predominantly focused on the direct effects of single factors on outcome variables, leaving the ‘black box’ of how pressure indirectly influences learning engagement largely unexplained. Therefore, by constructing a multiple mediation model, this study deeply analyzes the internal pathways through which perceived pressure affects learning engagement among Chinese doctoral students. This not only extends the application boundaries of the JD-R model in higher education but also provides detailed empirical evidence for understanding how doctoral students achieve psychological adaptation and academic development in pressure environments.

### 1.1 Mediating role of social support

Social support refers to the material and psychosocial assistance individuals obtain from their social networks when facing stressful events, primarily including emotional support and informational exchange (Cohen et al., 2004; Siedlecki et al., 2014). Within the framework of the Job Demands–Resources (JD-R) model, social support is conceptualized as an important contextual resource that can enhance learning motivation and engagement through the motivational process.

Previous research suggests a close association between perceived pressure and social support. According to conservation of resources theory, sustained high levels of pressure consume individuals’ limited psychological and temporal resources, thereby weakening their capacity to maintain and access social support (Hobfoll et al., 1990). In the context of doctoral training, prolonged pressure and its accompanying negative emotions may lead doctoral students to display irritability, withdrawal, or social avoidance, which can impair the quality of interactions with supervisors and peers and reduce their subjective perception of available social support (Sarason et al., 1990). When social support is compromised, doctoral students may find it more difficult to obtain emotional reassurance and academic guidance from their environment, which in turn may undermine their sustained engagement in learning and research activities.

In contrast, adequate and effective social support helps individuals maintain a positive psychological state and further Predicts learning engagement by strengthening self-efficacy (Bakker and Demerouti, 2007). In higher education settings, institutional support from universities and a positive academic climate have been shown to significantly enhance doctoral students’ learning engagement (Pyhältö et al., 2023). Empirical studies further indicate that, under high-pressure conditions, insufficient perceived social support is associated with a marked decline in engagement willingness, making individuals more prone to reduced involvement, psychological withdrawal, or even disengagement (Alarcon et al., 2011; Siu et al., 2021; Peltonen et al., 2017).

Social support represents not only a crucial external resource for doctoral students coping with academic pressure but also a potential mediating mechanism linking perceived pressure to learning engagement. Variations in pressure levels may influence doctoral students’ engagement by shaping the availability and perception of social support, thereby providing a theoretical basis for the mediating hypothesis of social support proposed in the present study.

### 1.2 Mediating role of academic self-efficacy

Self-efficacy refers to an individual’s judgment of their capabilities to successfully execute specific tasks (Bandura, 1997), while academic self-efficacy represents students’ subjective beliefs regarding their capabilities to achieve academic goals (Song and Hu, 2024). As a key personal resource, academic self-efficacy serves as a core driving force in the individual’s motivational process. Perceived pressure, as a significant contextual factor, exerts a notable influence on academic self-efficacy (Ma, 2025). Regarding this relationship, there are two distinct perspectives in the literature. One view argues that chronic or high-intensity perceived pressure depletes students’ cognitive and emotional resources, thereby diminishing their academic self-efficacy (Gao, 2023). For instance, Lu et al. (2025) verified a negative relationship between pressure and academic self-efficacy among Chinese elite university students and students preparing for the college entrance examination, respectively. Conversely, another view posits that increased perceived pressure can enhance students’ academic self-efficacy (Pintrich and Schunk, 1996).

Students’ learning engagement is largely contingent upon their academic self-efficacy (Pajares and Schunk, 2001). High levels of perceived pressure are often accompanied by negative emotions such as anxiety and fatigue, which directly diminish academic self-efficacy (Ma, 2025). Furthermore, academic self-efficacy determines the intensity of engagement individuals exhibit when completing academic tasks (Wang and Gao, 2021). Students with low academic self-efficacy tend to adopt avoidance strategies, struggle to exert effort in their studies, and exhibit greater passivity (Komarraju and Dial, 2014), or even experience academic burnout (Lu et al., 2025). In contrast, students with high academic self-efficacy demonstrate greater concentration, persistence, and initiative, thereby displaying superior levels of learning engagement (Wang and Gao, 2021).

### 1.3 Relationship between social support and academic self-efficacy

When examining the joint roles of doctoral students’ perceived pressure, social support, and academic self-efficacy in shaping learning engagement, Self-Determination Theory (SDT) provides a systematic framework for understanding how external support can be transformed into internal motivation and psychological resources. According to SDT, individuals’ motivation and engagement are not determined solely by external conditions or personal traits, but are jointly influenced by social support and internal psychological resources such as self-efficacy (Ryan and Deci, 2020). Within this framework, social support functions as a key external protective resource that satisfies individuals’ emotional and relational needs, facilitates the development of positive self-perceptions, and strengthens self-efficacy. In turn, higher levels of self-efficacy constitute an important psychological foundation for sustained learning engagement.

Empirical research further suggests that, in high-pressure contexts, social support not only exerts a direct motivational influence but may also operate indirectly by shaping academic self-efficacy. When doctoral students face elevated academic demands and pressure, support from supervisors, peers, and family can help buffer the erosion of self-beliefs caused by stress and enable students to maintain a positive evaluation of their academic capabilities. For example, a study of doctoral students in public administration found that social support from peers and family facilitated work–life balance, thereby enhancing academic self-efficacy and subsequently promoting research engagement (Schwoerer et al., 2021).

Based on the above theoretical and empirical evidence, it can be inferred that, within the high-demand context of doctoral training, perceived pressure may not influence learning engagement directly. Instead, perceived pressure may shape the availability and perception of social support, which subsequently enhances academic self-efficacy and, in turn, affects learning engagement. This process reflects a pathway through which external environmental resources are transformed into internal psychological resources, providing a clear rationale for the proposed chain mediation involving social support and academic self-efficacy.

### 1.4 Present study and hypotheses

**Figure 1:**
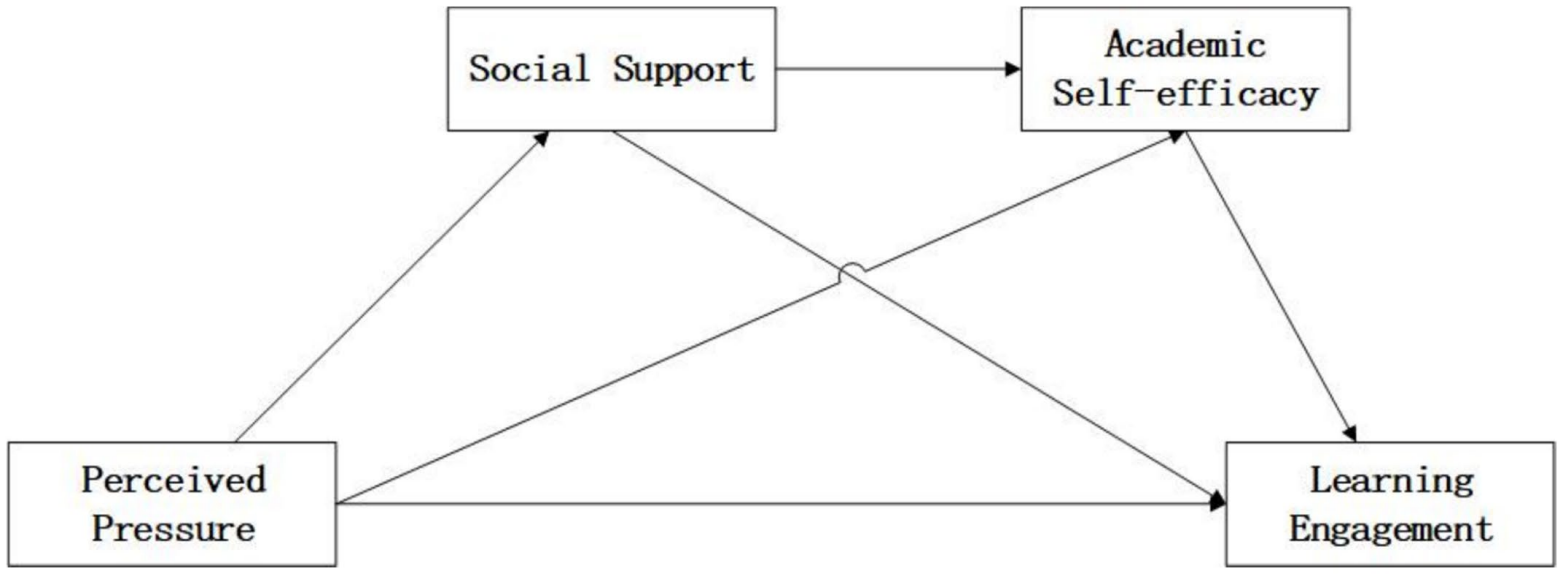
Hypothetical model of the influence of perceived pressure and learning engagement.

Existing research indicates that bivariate relationships exist among perceived pressure, learning engagement, social support, and academic self-efficacy. However, few studies have investigated their systematic impact on doctoral students’ learning engagement, and the potential mediating roles of social support and academic self-efficacy remain insufficiently explored. Therefore, grounded in the Job Demands–Resources model, this study investigates the roles of social support and academic self-efficacy in the process of transforming doctoral students’ perceived pressure into sustained learning engagement (see Figure 1).

We propose the following directionally specified hypotheses (the hypothetical model is shown in Figure 1):

*H1*: The perceived pressure of doctoral students affects their learning engagement.

*H2*: Social support plays a mediating role in the relationship between doctoral students' perceived pressure and learning engagement.

*H3*: Academic self-efficacy plays a mediating role in the relationship between doctoral students' perceived pressure and learning engagement.

*H4*: Social support and academic self-efficacy play a chain-mediated role in the effects of doctoral students' perceived pressure on learning engagement.

## 2 Design

### 2.1 Participants

To obtain the data, the author searched core journals across disciplines in the Chinese Social Science Citation Index (CSSCI)—the authoritative journal evaluation system in China—and collected email addresses of doctoral students who had published papers in these journals over the past 5 years. Data collection spanned from April to August 2025. In total, 700 doctoral students were identified and invited to participate; 500 questionnaires were returned, of which 432 were valid, yielding a validity response rate of 86.4%. Regarding sample composition, the respondents originally spanned all 13 official disciplinary categories defined by the Chinese Ministry of Education. To ensure conciseness and theoretical clarity, these disciplines were grouped into four distinct categories based on Becher's (1989) typology. Furthermore, the sample demonstrates strong structural diversity, covering doctoral students from both research-oriented and applied universities across more than 20 provinces and municipalities in China. Detailed sample characteristics are presented in Table 1.

**Table 1:** The demographics of the doctoral students.

Variables		Category	Frequency (*n* = 432)	Ratio
Control variable	Gender	Male	285	65.972
		Female	147	34.028
	Types	Academic	288	66.667
		Professional	144	33.333
	Major	Hard-Pure	34	7.870
		Soft-Pure	205	47.454
		Hard-Applied	85	19.676
		Soft-Applied	108	25
	Age	Age 20–30	222	51.389
		Age 30–40	177	40.972
		Age 40–50	31	7.176
		Over 50	2	0.463
	Grade	First year of PhD	56	12.963
		Second year of PhD	139	32.176
		Third year of PhD	138	31.944
		PhD 4th year and above	99	22.917
	Annual Family Income	10,000–30,000 RMB	25	5.787
		30,000–60,000 RMB	85	19.676
		60,000–100,000 RMB	126	29.167
		100,000–200,000 RMB	131	30.324
		200,000–500,000 RMB	45	10.417
		More than 500,000 RMB	20	4.630

### 2.2 Method

#### 2.2.1 Perceived pressure scale

In this study, the Graduate Stress Scale (GSS) was used to measure the perceived pressure level of graduate students (Rocha-Singh, 1994). The scale was specifically designed to evaluate the pressure of graduate students, including environmental (e.g., Lack of same race/ethnicity partners on campus), family (e.g., Family having health problems), and academic pressures (e.g., Fear of failure in the program expectations). 21 items are rated on a seven-point scale, ranging from 1 (lowest intensity) to 7 (highest intensity). In the current sample, the Cronbach’s *α* was 0.942.

#### 2.2.2 Learning engagement scale

Learning engagement was measured using the Classroom Engagement Scale (Skinner et al., 2008), which comprises two core dimensions: behavioral engagement (e.g., Attention, Concentration) and emotional engagement (e.g., Enthusiasm). 14 items are rated on a four-point scale, ranging from 1 (not engaged at all) to 4 (very engaged). Classical theories often separate learning engagement into three domains—cognitive, behavioral, and emotional. The present study adopts the bipartite conceptualization rooted in the Motivational Model of Engagement. Within this theoretical framework, engagement is strictly defined as the outward manifestation of motivation, characterized by behavioral participation and emotional quality. We excluded the cognitive dimension due to its conceptual overlap with self-regulation and coping mechanisms, as well as measurement redundancies with emotional components (Fredricks et al., 2016). Furthermore, this structure is particularly well-suited for the doctoral context: unlike undergraduates who rely on structured cognitive tasks, doctoral engagement is predominantly defined by the persistence to conduct independent research and the affective resilience against academic pressure. In the current sample, Cronbach’s *α* for the scale was 0.924.

#### 2.2.3 Social support scale

Social support was assessed using the Social Support Rating Scale (SSRS) developed by Xiao (1994). The scale comprises 10 items across three dimensions: objective support, subjective support, and support utilization. An example item is “How many close friends do you have who can provide you with support and help?” In this study, the scoring criteria were modified to suit the research context: Items 1–4 and 8–10 retained the original 4-point scale, while Items 5, 6, and 7 were adapted into a multiple-response format where each selected option contributed 1 point, capped at a maximum of 4 points per item. The total score is the summation of all item scores, with higher totals indicating greater social support. The scale has demonstrated robust reliability in Chinese graduate populations, and Cronbach’s *α* for the current sample was 0.821.

#### 2.2.4 Academic self-efficacy scale

Academic self-efficacy was measured using the General Academic Self-Efficacy Scale (Nielsen et al., 2018). This scale consists of five items rated on a five-point Likert scale, ranging from 1 (very poor) to 5 (very good). The total score is the sum of all item scores, with higher totals indicating greater academic self-efficacy. An example item is “I generally manage to solve difficult academic problems if I try hard enough.” The scale has demonstrated robust psychometric properties in cross-cultural validation studies of American, Belgian, and Dutch college students (van Zyl et al., 2022). In the current sample, the Cronbach’s α coefficient of the scale was 0.855, indicating good reliability.

### 2.3 Control variable

Studies indicate that doctoral students’ gender (Xin et al., 2024), grade level (Moreira et al., 2023), age (Nja et al., 2023), and family economic status (Zhu, 2023) significantly influence perceived pressure or learning engagement. To mitigate potential confounding effects, these demographic factors were included as control variables in the analysis.

### 2.4 Data analysis

First, data screening was performed to exclude questionnaires exhibiting response sets or logical inconsistencies. Next, descriptive statistics were computed for demographic characteristics and core variables. Pearson correlation analysis was then conducted to examine the bivariate associations among these variables. Finally, for mediation analyzes, the regression-based approach recommended by Hayes and Rockwood (2017) was adopted. Specifically, Models 4 and 6 of the PROCESS macro for SPSS (Version 24.0) were utilized, with gender, age, grade, and family income entered as covariates to control for their potential confounding effects. Model 4 was used to examine the independent mediating effects of social support and academic self-efficacy between perceived pressure and learning engagement, Model 6 was employed to test the chain mediation effect. The significance of indirect effects was assessed using the bias-corrected percentile bootstrap method with 5,000 resamples to generate 95% confidence intervals (CIs).

## 3 Results

### 3.1 Measurement model testing

Since the data were derived from self-reported measures, the potential for common method bias (CMB) was addressed through both procedural and statistical means. Procedurally, measures such as respondent anonymity and randomized item ordering were implemented during data collection to minimize bias. Statistically, we first rigorously verified the construct validity of the measurement model using Confirmatory Factor Analysis (CFA) in AMOS 24.0. The results indicated that the four-factor measurement model (Perceived Pressure, Social Support, Academic Self-Efficacy, and Learning Engagement) fitted the data satisfactorily (χ^2^/df = 1.106, RMSEA = 0.016, RMR = 0.025, IFI = 0.997, TLI = 0.996, CFI = 0.997).

Furthermore, convergent validity was assessed (see Table 2). While the Average Variance Extracted (AVE) values for some constructs were slightly below the 0.50 threshold, the Composite Reliability (CR) values for all constructs ranged from 0.796 to 0.939, well above the recommended cutoff of 0.60. According to Fornell and Larcker (1981), if AVE is less than 0.5 but CR is higher than 0.6, the convergent validity of the construct is still considered adequate.

**Table 2:** Reliability and validity of the constructs.

Factor	AVE	CR
Perceived pressure	0.424	0.939
Social support	0.329	0.796
Academic self-efficacy	0.544	0.856
Learning engagement	0.484	0.926

Following this validation, Harman’s single-factor test was conducted. The results extracted nine factors with eigenvalues exceeding 1, with the first factor accounting for only 28.343% of the total variance. This is well below the 40% threshold (Podsakoff et al., 2003), suggesting that common method bias is not a pervasive issue in this study.

### 3.2 Descriptive statistics and correlation analysis

Table 3 presents the descriptive statistics and Pearson correlation coefficients for all research variables. As the data indicate, perceived pressure exhibited significant positive correlations with social support (*r* = 0.379, *p* < 0.01), academic self-efficacy (*r* = 0.394, *p* < 0.01), and learning engagement (*r* = 0.414, *p* < 0.01). Furthermore, social support was positively associated with both academic self-efficacy (*r* = 0.36, *p* < 0.01) and learning engagement (*r* = 0.395, *p* < 0.01). Finally, a robust positive relationship was observed between academic self-efficacy and learning engagement (*r* = 0.424, *p* < 0.01).

**Table 3:** Descriptive statistics and correlation coefficients between key variables (*N* = 432).

Variables	Max	Min	Mean	SD	1	2	3	4
1. Perceived pressure	7	1	4.660	0.840	1	-	-	-
2. Social support	4	1	2.712	0.564	0.379**	1	-	-
3. Academic self-efficacy	5	1	3.409	0.936	0.394**	0.36**	1	-
4. Learning engagement	4	1	3.464	0.528	0.414**	0.395**	0.424**	1

** indicates significance level *p* < 0.01.

### 3.3 Bootstrap analysis of mediating effect significance test

#### 3.3.1 Mediating effect of social support between perceived pressure and learning engagement

To examine the mediating role of social support in the relationship between perceived pressure and learning engagement, this study employed Model 4 of the PROCESS macro for SPSS (Hayes and Rockwood, 2017). A bias-corrected bootstrap procedure with 5,000 resamples was applied to estimate confidence intervals.

As presented in Table 4, the total effect of perceived pressure on learning engagement was significant (*β* = 0.260, *p* < 0.001). The results of the regression analysis indicated that perceived pressure significantly predicted social support (*β* = 0.255, *p* < 0.001), which in turn significantly predicted learning engagement (*β* = 0.256, *p* < 0.001). The indirect effect was estimated at 0.065. The 95% confidence interval [0.036, 0.100] did not include zero, confirming a significant mediation effect. Consequently, social support partially mediates the relationship between perceived pressure and learning engagement, accounting for 24.986% of the total effect.

**Table 4:** Analysis of the mediating effect of social support between perceived pressure and learning engagement.

Impact path	Meaning	Effect	SE	*p*	LLCL	ULCL
PS → SS → LE	Indirect Effect	0.065	0.025	*p* < 0.001	0.036	0.100
PS → SS		0.255	0.032	*p* < 0.001	0.196	0.313
SS → LE		0.256	0.043	*p* < 0.001	0.172	0.340
PS → LE	Direct Effect	0.195	0.030	*p* < 0.001	0.139	0.252
PS → LE	Total Effect	0.260	0.029	*p* < 0.001	0.206	0.315

PS, Perceived Pressure; SS, Social Support; LE, Learning Engagement; LLCL, Lower Limit of Confidence Interval; ULCL, Upper Limit of Confidence Interval.

#### 3.3.2 Mediating effect of academic self-efficacy between perceived pressure and learning engagement

As shown in Table 5, the total effect of perceived pressure on learning engagement was significant (*β* = 0.260, *p* < 0.001). The regression results indicated that perceived pressure significantly predicted academic self-efficacy (*β* = 0.435, *p* < 0.001), which in turn significantly predicted learning engagement (*β* = 0.172, *p* < 0.001). The indirect effect was estimated at 0.075. The 95% confidence interval [0.068, 0.173] did not include zero, confirming a significant mediation effect. Consequently, academic self-efficacy partially mediates the relationship between perceived pressure and learning engagement, accounting for 28.712% of the total effect.

**Table 5:** Analysis of the mediating effect of academic self-efficacy between perceived pressure and learning engagement.

Impact path	Effect	Effect	SE	*p*	LLCL	ULCL
PS → ASE → LE	Indirect Effect	0.075	0.027	*p* < 0.001	0.068	0.173
PS → ASE		0.435	0.049	*p* < 0.001	0.337	0.532
ASE → LE		0.172	0.026	*p* < 0.001	0.122	0.222
PS → LE	Direct Effect	0.186	0.029	*p* < 0.001	0.130	0.242
PS → LE	Total Effect	0.260	0.028	*p* < 0.001	0.206	0.315

PS, Perceived Pressure; ASE, Academic Self-Efficacy; LE, Learning Engagement; LLCL, Lower Limit of Confidence Interval; ULCL, Upper Limit of Confidence Interval.

#### 3.3.3 Chain mediating effects of social support and academic self-efficacy

Finally, this study examined whether social support and academic self-efficacy exert a chain mediating effect on the relationship between perceived pressure and learning engagement (see in Table 6). The results indicated that the indirect path (Perceived Pressure → Social Support→Academic Self-Efficacy →Learning Engagement) was statistically significant. The effect size was 0.015, with a 95% bias-corrected confidence interval of [0.010, 0.041]. Since this interval excludes zero, the chain mediation effect is significant (*p* < 0.05).

**Table 6:** Analysis of the chain mediating effects of social support and academic self-efficacy between perceived pressure and learning engagement.

Impact path	Effect	SE	*p*	LLCL	ULCL
PS → SS → ASE → LE	0.015	0.008	*p* < 0.05	0.010	0.041

PS, Perceived pressure; ASE, Academic Self-Efficacy; LE, Learning Engagement; LLCL, Lower Limit of Confidence Interval; ULCL, Upper Limit of Confidence Interval.

## 4 Discussion

Within the framework of the Job Demands–Resources (JD-R) model, this study elucidated the mechanisms linking doctoral students’ perceived pressure to their learning engagement, with particular attention paid to the serial mediating roles of social support and academic self-efficacy. The results revealed that perceived pressure is not only associated with learning engagement but also fosters learning engagement indirectly through the independent and chained mediation of social support and academic self-efficacy.

At the theoretical level, this study dissects the psychological pathways through which perceived pressure translates into learning engagement. By identifying the sequential mediating roles of social support and academic self-efficacy, this research extends the explanatory boundaries of the JD-R model regarding the pressure–adaptation process in higher education. Departing from prior studies that often pathologize pressure as merely a risk factor (Kort-Butler, 2019; Malykhin et al., 2025), our findings imply that in resource-rich environments, pressure can function as a catalyst. Through a mechanism of resource activation, environmental demands stimulate the mobilization of social and psychological resources, thereby driving sustained learning engagement.

At the practical level, the findings advocate for holistic interventions in doctoral training that integrate pressure management with resource building. Specifically, universities and supervisors should prioritize the construction of multi-tiered social support ecosystems and the fortification of academic self-efficacy. These strategies are essential for empowering doctoral students to transmute the pressure of high-stakes environments into productive learning engagement, thereby enhancing the overall quality of doctoral education.

### 4.1 Current situation

Based on the descriptive statistics, the doctoral students in this sample generally exhibit a profile characterized by “moderate pressure–high resources–high engagement.” As shown in Table 2, the mean score for perceived pressure was 4.660, aligning with the theoretical midpoint of 4, which indicates a moderate level of pressure. The mean score for social support was 2.712 (slightly above the theoretical midpoint of 2.5), suggesting that participants generally perceive a certain level of supportive resources. The mean score for academic self-efficacy was 3.409 (notably higher than the theoretical midpoint of 3), demonstrating a strong sense of academic competence and self-belief. Furthermore, the mean score for learning engagement was 3.464 (significantly exceeding the theoretical midpoint of 2.5), indicating that doctoral students in this study generally maintain high levels of academic participation and engagement.

Correlation analysis further revealed the interplay among these variables. Perceived pressure showed significant positive correlations with social support, academic self-efficacy, and learning engagement (*p* < 0.01). Interpreted through the lens of the Job Demands–Resources (JD-R) model, this suggests that while doctoral students endure prominent pressure, they are simultaneously able to mobilize relatively abundant social support and internal psychological resources. Consequently, for many doctoral students, this pressure functions more as a “challenge stressor,” thereby stimulating learning engagement.

### 4.2 Direct influence of perceived pressure on learning engagement

The study found that perceived pressure serves as a significant positive predictor of learning engagement (*β* = 0.414, *p* < 0.001). Specifically, for every one standard deviation increase in perceived pressure, learning engagement increases by 0.414 standard deviations. This result suggests that within the current sample, perceived pressure does not merely manifest as a ‘risk factor’ suppressing learning engagement. This finding aligns with existing research indicating that moderate stress contributes to enhanced cognitive function and learning performance. For instance, studies by Vogel and Schwabe (2016) and Zoladz et al. (2011) have demonstrated that moderate levels of stress can, under certain conditions, enhance memory processing and task engagement. Focusing on the doctoral cohort, this study reaffirms the positive role of perceived pressure within high-demand academic contexts.

This stands in contrast to previous conclusions, which highlighted that the prevalence of mental health issues like depression and anxiety among doctoral students exerts detrimental effects on academic performance and persistence (Levecque et al., 2017). Our findings suggest that the impact of perceived pressure is not simply ‘the higher, the worse.’ Its function depends largely on the stress level and the resources available to the individual (Chen et al., 2024). In contexts where resources are relatively abundant, moderate perceived pressure can foster doctoral students’ learning engagement by stimulating effort and a sense of responsibility.

It is important to acknowledge that the Graduate Stress Scale (GSS) used in this study primarily measures the intensity of stress exposure rather than the students’ cognitive appraisal of stress (i.e., whether they view it as a challenge or a hindrance). However, our findings show a positive association between perceived pressure and engagement, suggesting that in the context of high resource availability (as observed in our sample), these high-intensity demands are functionally operating as ‘challenge demands’ that stimulate effort, rather than ‘hindrance demands’ that deplete it. Future research should explicitly measure stress appraisal to confirm this mechanism.

### 4.3 Mediating role of social support and academic self-efficacy

Regarding the pathway mediated by social support, learning engagement is positively related to perceived pressure by elevating social support levels. This indirect effect accounts for 24.986% of the total effect, underscoring the significant mediating role of social support in the pressure–engagement relationship. This finding aligns with prior research demonstrating the mediating function of social support in the link between stress and work engagement (Hakanen et al., 2008). Notably, this study reveals a distinctive pattern: doctoral students experiencing higher pressure did not succumb to withdrawal or psychological exhaustion. Instead, in the face of intensified pressure, they were more inclined to mobilize social support networks to compensate for personal resource deficits, thereby sustaining their learning engagement. In other words, heightened perceived pressure may serve to activate the individual’s support system. Concurrently, a stronger social support network bolsters confidence in coping with academic challenges, thereby increasing the likelihood of successful degree completion and sustained learning engagement. This corroborates the conclusion of García-Machado et al. (2024) that, under conditions of resource sufficiency, moderate pressure can enhance behavioral efficiency and goal persistence via social support, ultimately maintaining high levels of learning engagement within stressful contexts.

In the pathway mediated by academic self-efficacy, perceived pressure is positively related to learning engagement by bolstering academic self-efficacy levels. The indirect effect was estimated at 0.075, accounting for 28.712% of the total effect. This finding resonates with the core tenets of Job Demands–Resources (JD-R) theory: in high-demand contexts, provided individuals can leverage sufficient resources, perceived pressure can be transformed into a driver of engagement through a motivational process. As a critical personal resource, academic self-efficacy acts as a pivot in this process. On the one hand, the pressure context stimulates coping motivation, prompting doctoral students to accumulate mastery experiences through overcoming difficulties, thereby enhancing academic confidence. On the other hand, this fortified self-efficacy further strengthens goal commitment and perceived task control, enabling the maintenance of high levels of focus and effort even within high-pressure environments.

### 4.4 The chain mediating effect of social support and academic self-efficacy

The chain mediation analysis further elucidates how perceived pressure influences doctoral students’ learning engagement through the interplay of external social resources and internal psychological resources. Specifically, perceived pressure prompts doctoral students to actively mobilize external social resources to cope with challenges. The utilization of this social support is then converted into internal academic self-efficacy, thereby enhancing students’ confidence in their research and problem-solving capabilities. Ultimately, this heightened self-efficacy drives students to maintain deep learning engagement—even under high-pressure conditions—by strengthening goal commitment and persistence. This forms a complete transmutation pathway: ‘Perceived Pressure→Social Support→Academic Self-Efficacy→Learning Engagement.’

It is worth noting that the effect size of the chain mediation path was significantly smaller than that of the single mediation paths. This phenomenon is primarily attributed to the statistical ‘cumulative attenuation effect’: as the mediation chain lengthens, the influence of the predictor variable is inevitably diluted during the multi-stage transmission process. Furthermore, this reflects the incompleteness of resource conversion. Although perceived pressure can motivate individuals to acquire external social support, this support does not automatically or fully internalize into academic self-efficacy. This process is constrained by individual cognitive processing and other factors, thereby limiting the overall strength of the chain mediation effect.

## 5 Conclusion

This study elucidates how doctoral students’ perceived pressure influences their learning engagement. Perceived pressure not only directly increases engagement but also operates indirectly through two independent mediators—social support and academic self-efficacy—and through a chained mediation pathway: social support → academic self-efficacy. These findings refine our understanding of the pressure–adaptation process and show that, given adequate resources, perceived pressure can be transformed into academic motivation. The results imply that universities should implement an integrated support system that combines pressure management, social support, and self-efficacy enhancement. By building multi-level support networks and designing stepped academic tasks, universities can help doctoral students transform stressful experiences into positive drivers of academic development and thereby improve the quality of doctoral training.

This study has several limitations that should be addressed in future research. First, the sample consisted entirely of doctoral students who had published papers in CSSCI journals within the past 5 years; this high-performing group may have advantages in research ability, learning engagement, and access to resources, and therefore may not fully represent the overall doctoral student population, limiting the generalizability of the findings. Second, the cross-sectional design represents a fundamental limitation. While the serial mediation model implies a directional process, our data only capture concurrent associations. It is plausible that highly engaged students perceive less pressure or attract more social support (reverse causality). Therefore, the causal directions proposed in our model are theoretical and rely on the JD-R framework; they cannot be empirically proven without longitudinal or experimental data.

## Data Availability

The original contributions presented in the study are included in the article/supplementary material, further inquiries can be directed to the corresponding author.

## References

[ref1] Alarcon G. M. Edwards J. M. Menke L. E. (2011). Student burnout and engagement: a test of the conservation of resources theory. J. Psychol. 145, 211–227. doi: 10.1080/00223980.2011.555432, 21560805

[ref2] Bakker A. B. Demerouti E. (2007). The job demands–resources model: state of the art. J. Manage. Psychol. 22, 309–328. doi: 10.1108/02683940710733115

[ref4] Bandura A. (1997). Self-efficacy: The exercise of control. New York, NY: Freeman.

[ref5] Becher T. (1989). Academic tribes and territories: Intellectual enquiry and the culture of disciplines. Milton Keynes: Open University Press.

[ref6] Byrom N. C. Dinu L. Kirkman A. Hughes G. (2020). Predicting stress and mental wellbeing among doctoral researchers. J. Ment. Health 31, 783–791. doi: 10.1080/09638237.2020.1818196, 32967498

[ref7] Casey C. Taylor J. Knight F. Trenoweth S. (2023). Understanding the mental health of doctoral students. Encyclopedia 3, 1523–1536. doi: 10.3390/encyclopedia3040109

[ref8] Chen Y. Li C. Cao L. Liu S. (2024). The effects of self-efficacy, academic stress, and learning behaviors on self-regulated learning in blended learning among middle school students. Educ. Inf. Technol. 29, 24087–24110. doi: 10.1007/s10639-024-12821-w

[ref9] Cohen A. N. Hammen C. Henry R. M. Daley S. E. (2004). Effects of stress and social support on recurrence in bipolar disorder. J. Affect. Disord. 82, 143–147. doi: 10.1016/j.jad.2003.10.008, 15465589

[ref10] Demerouti E. Bakker A. B. Nachreiner F. Schaufeli W. B. (2001). The job demands-resources model of burnout. J. Appl. Psychol. 86, 499–512. doi: 10.1037/0021-9010.86.3.499 11419809

[ref11] Desie Y. Tefera B. (2017). Doctoral students’ academic engagements in Addis Ababa university, Ethiopia: nature, sources, and challenges. Int. J. Afr. High. Educ. 4:10251. doi: 10.6017/ijahe.v4i1.10251

[ref12] Fornell C. Larcker D. F. (1981). Evaluating structural equation models with unobservable variables and measurement error. J. Mark. Res. 18, 39–50. doi: 10.1177/002224378101800104

[ref13] Fredricks J. A. Filsecker M. Lawson M. A. (2016). Student engagement, context, and adjustment: addressing definitional, measurement, and methodological issues. Learn. Instr. 43, 1–4. doi: 10.1016/j.learninstruc.2016.02.002

[ref14] Gao X. (2023). Academic stress and academic burnout in adolescents: a moderated mediating model. Front. Psychol. 14:3706. doi: 10.3389/fpsyg.2023.1133706, 37342640 PMC10278958

[ref15] García-Machado J. J. Martínez Ávila M. Dospinescu N. Dospinescu O. (2024). How the support that students receive during online learning influences their academic performance. Educ. Inf. Technol. 29, 20005–20029. doi: 10.1007/s10639-024-12639-6

[ref16] Girard N. Cardona A. Fiorelli C. (2024). Learning how to develop a research question throughout the PhD process: training challenges, objectives, and scaffolds drawn from doctoral programs for students and their supervisors. High. Educ. 89, 1001–1020. doi: 10.1007/s10734-024-01258-2

[ref17] Goodnite P. M. (2013). Stress: a concept analysis. Nurs. Forum 49, 71–74. doi: 10.1111/nuf.12044 24456555

[ref19] Hakanen J. J. Schaufeli W. B. Ahola K. (2008). The job demands–resources model: a three-year cross-lagged study of burnout, depression, commitment, and work engagement. Work Stress. 22, 224–241. doi: 10.1080/02678370802379432

[ref20] Hayes A. F. Rockwood N. J. (2017). Regression-based statistical mediation and moderation analysis in clinical research: observations, recommendations, and implementation. Behav. Res. Ther. 98, 39–57. doi: 10.1016/j.brat.2016.11.001, 27865431

[ref21] Hobfoll S. E. Freedy J. Lane C. Geller P. (1990). Conservation of social resources: social support resource theory. J. Soc. Pers. Relat. 7, 465–478. doi: 10.1177/0265407590074004

[ref22] Horan K. A. Nakahara W. H. DiStaso M. J. Jex S. M. (2020). A review of the challenge-hindrance stress model: recent advances, expanded paradigms, and recommendations for future research. Front. Psychol. 11:560346. doi: 10.3389/fpsyg.2020.560346, 33224054 PMC7674200

[ref23] Komarraju M. Dial C. (2014). Academic identity, self-efficacy, and self-esteem predict self-determined motivation and goals. Learn. Individ. Differ. 32, 1–8. doi: 10.1016/j.lindif.2014.02.004

[ref24] Kort-Butler L. A. (2019). “The stress mechanisms of adolescent physical, mental, and Behavioral health” in Routledge international handbook of delinquency and health. eds. Vaughn M. G. Salas-Wright C. P. Jackson D. B. (New York: Routledge), 74–89.

[ref25] Levecque K. Anseel F. De Beuckelaer A. Van der Heyden J. Gisle L. (2017). Work organization and mental health problems in PhD students. Res. Policy 46, 868–879. doi: 10.1016/j.respol.2017.02.008

[ref26] Lu C. Chen J. Xie J. Qian Y. Fan Z. Peng D. . (2025). Effects of physical activity on academic burnout among rural left-behind children in China: the chain-mediated roles of loneliness and general self-efficacy. Front. Psychol. 16:1653243. doi: 10.3389/fpsyg.2025.1653243 41209803 PMC12589049

[ref27] Ma X. (2025). The relationship between psychological stress and academic performance among college students: the mediating roles of cognitive load and self-efficacy. Acta Psychol. 259:105433. doi: 10.1016/j.actpsy.2025.105433, 40829194

[ref28] Mahsood N. Mahboob U. Asim M. Shaheen N. (2025). Assessing the well-being of PhD scholars: a scoping review. BMC Psychol. 13:362. doi: 10.1186/s40359-025-02668-2, 40211401 PMC11984241

[ref29] Malykhin N. Serrano J. Pietrasik W. Hegadoren K. (2025). Effects of duration and intensity of psychological stressors on mental health outcomes. J. Psychiatr. Res. 187, 211–222. doi: 10.1016/j.jpsychires.2025.05.012, 40382943

[ref30] Mavrogalou-Foti A. P. Kambouri M. A. Çili S. (2024). The supervisory relationship as a predictor of mental health outcomes in doctoral students in the United Kingdom. Front. Psychol. 15:1437819. doi: 10.3389/fpsyg.2024.1437819, 39444829 PMC11497167

[ref31] McAlpine L. Amundsen C. (2011). Doctoral education: Research-based strategies for doctoral students, supervisors and administrators. Netherlands: Springer.

[ref32] McCray J. Joseph-Richard P. (2020). Towards a model of resilience protection: factors influencing doctoral completion. High. Educ. 80, 679–699. doi: 10.1007/s10734-020-00507-4

[ref33] Moreira T. Martins J. Núñez J. C. Oliveira A. Martins J. Rosário P. (2023). Acculturation and school engagement: the case of Portuguese students with Roma background. Rev. Psicodidact. 28, 67–79. doi: 10.1016/j.psicoe.2022.11.003

[ref35] Nielsen T. Dammeyer J. Vang M. L. Makransky G. (2018). Gender fairness in self-efficacy? A Rasch-based validity study of the general academic self-efficacy scale (GASE). Scand. J. Educ. Res. 62, 664–681. doi: 10.1080/00313831.2017.1306796

[ref36] Nja C. O. Anari M. I. Erim C. M. Idiege K. J. Ilhami A. Ukah J. U. . (2023). Learning space, students’ collaboration, educational outcomes, and interest: exploring the physical, social and psychological mediators. Heliyon 9:e15456. doi: 10.1016/j.heliyon.2023.e15456, 37123935 PMC10131041

[ref37] Pajares F. Schunk D. (2001). “The development of academic self-efficacy” in Development of achievement motivation. eds. Wigfield A. Eccles J. S. (San Diego, CA: Academic Press), 15–31.

[ref38] Peltonen J. A. Vekkaila J. Rautio P. Haverinen K. Pyhältö K. (2017). Doctoral students' social support profiles and their relationship to burnout, drop-out intentions, and time to candidacy. Int. J. Doctoral Stud. 12, 157–173. doi: 10.28945/3792

[ref40] Pintrich P. R. Schunk D. H. (1996). Motivation in education: Theory, research, and applications. Englewood Cliffs, NJ: Merrill/Prentice Hall.

[ref41] Podsakoff N. P. LePine J. A. LePine M. A. (2007). Differential challenge stressor–hindrance stressor relationships with job attitudes, turnover intentions, turnover, and withdrawal behavior: a meta-analysis. J. Appl. Psychol. 92, 438–454. doi: 10.1037/0021-9010.92.2.438, 17371090

[ref42] Podsakoff P. M. MacKenzie S. B. Lee J. Y. Podsakoff N. P. (2003). Common method biases in behavioral research: a critical review of the literature and recommended remedies. J. Appl. Psychol. 88, 879–903. doi: 10.1037/0021-9010.88.5.879, 14516251

[ref43] Pyhältö K. Peltonen J. Anttila H. Frick L. L. de Jager P. (2023). Engaged and/or burnt out? Finnish and south African doctoral students’ experiences. Stud. Grad. Postdoctor. Educ. 14, 1–18. doi: 10.1108/sgpe-02-2021-0013

[ref44] Rocha-Singh I. A. (1994). Perceived pressure among graduate students: development and validation of the graduate stress inventory. Educ. Psychol. Meas. 54, 714–727. doi: 10.1177/0013164494054003018

[ref45] Ryan R. M. Deci E. L. (2020). Intrinsic and extrinsic motivation from a self-determination theory perspective: definitions, theory, practices, and future directions. Contemp. Educ. Psychol. 61:101860. doi: 10.1016/j.cedpsych.2020.101860

[ref46] Sarason I. G. Sarason B. R. Pierce G. R. (1990). Social support: the search for theory. J. Soc. Clin. Psychol. 9:133. doi: 10.1521/jscp.1990.9.1.133

[ref47] Schwoerer K. Antony M. Willis K. (2021). #PhDlife: the effect of stress and sources of support on perceptions of balance among public administration doctoral students. J. Public Aff. Educ. 27, 326–347. doi: 10.1080/15236803.2021.1876474

[ref48] Siedlecki K. L. Salthouse T. A. Oishi S. Jeswani S. (2014). The relationship between social support and subjective well-being across age. Soc. Indic. Res. 117, 561–576. doi: 10.1007/s11205-013-0361-4, 25045200 PMC4102493

[ref49] Siu O. L. Lo B. C. Y. Ng T. K. Wang H. (2021). Social support and student outcomes: the mediating roles of psychological capital, study engagement, and problem-focused coping. Curr. Psychol. 42, 2670–2679. doi: 10.1007/s12144-021-01621-x

[ref50] Skinner E. Furrer C. Marchand G. Kindermann T. (2008). Engagement and disaffection in the classroom: part of a larger motivational dynamic? J. Educ. Psychol. 100:765. doi: 10.1037/a0012840

[ref51] Song X. Hu Q. (2024). The relationship between freshman students’ mental health and academic achievement: chain mediating effect of learning adaptation and academic self-efficacy. BMC Public Health 24:3207. doi: 10.1186/s12889-024-20738-9, 39558273 PMC11575212

[ref52] Stillwell S. B. Vermeesch A. L. Scott J. G. (2017). Interventions to reduce perceived pressure among graduate students: a systematic review with implications for evidence-based practice. Worldviews Evid.-Based Nurs. 14, 507–513. doi: 10.1111/wvn.12250, 28795775

[ref53] van Zyl L. E. Klibert J. Shankland R. See-To E. W. Rothmann S. (2022). The general academic self-efficacy scale: psychometric properties, longitudinal invariance, and criterion validity. J. Psychoeduc. Assess. 40, 777–789. doi: 10.1177/07342829221097174

[ref54] Vogel S. Schwabe L. (2016). Learning and memory under stress: implications for the classroom. NPJ Sci. Learn. 1, 1–10. doi: 10.1038/npjscilearn.2016.11, 30792896 PMC6380371

[ref55] Wang L. H. Gao Y. Y. (2021). Achievement goal orientation for postgraduates and academic procrastination at research universities: the mesomeric effect of academic self-efficacy. J. Grad. Educ. 3, 26–34. doi: 10.19834/j.cnki.yjsjy2011.2021.03.05

[ref56] Weerasinghe S. P. S. P. (2024). Perceived pressure and sources of stress among university academics during the COVID-19 pandemic. Int. J. Res. Innov. Soc. Sci. 8, 2126–2136. doi: 10.47772/ijriss.2024.8090176

[ref57] Woolston C. O’Meara S. (2019). PhD students in China report misery and hope. Nature 575, 711–713. doi: 10.1038/d41586-019-03631-z, 31772365

[ref58] Xiao S. (1994). Theoretical basis and research application of social support rating scale. J. Clin. Psychiatry 2, 98–100.

[ref59] Xin M. Yang C. Zhang L. Gao C. Wang S. (2024). The impact of perceived life stress and online social support on university students’ mental health during the post-COVID era in northwestern China: gender-specific analysis. BMC Public Health 24:467. doi: 10.1186/s12889-024-17935-x, 38355474 PMC10868037

[ref60] Yang M. Yuan R. (2020). Early-stage doctoral students’ conceptions of research in higher education: cases from Hong Kong. Teach. High. Educ. 28, 85–100. doi: 10.1080/13562517.2020.1775191

[ref61] Zhu Y. (2023). A study on the effect of family capital on college students’ academic achievement. Lec.t Note Educ. Psychol. Public Media 16, 15–19. doi: 10.54254/2753-7048/16/20231095

[ref62] Zoladz P. R. Clark B. Warnecke A. Smith L. Tabar J. Talbot J. N. (2011). Pre-learning stress differentially affects long-term memory for emotional words, depending on temporal proximity to the learning experience. Physiol. Behav. 103, 467–476. doi: 10.1016/j.physbeh.2011.01.016, 21262248

